# Nonfemoral Arterial Hemostasis Following Percutaneous Intervention Using a Focused Compression Device

**DOI:** 10.1007/s00270-020-02431-7

**Published:** 2020-02-10

**Authors:** Louis-Xavier Barrette, Ansar Z. Vance, Susan Shamimi-Noori, Gregory P. Nadolski, Shilpa Reddy, Kathleen M. Kratz, Jonas W. Redmond, Timothy W. I. Clark

**Affiliations:** Section of Interventional Radiology, Department of Radiology, Penn Presbyterian Medical Center, Perelman School of Medicine of the University of Pennsylvania, Philadelphia, PA 19104 USA

**Keywords:** Radial artery access, Hemostasis device, Peripheral vascular intervention

## Abstract

**Purpose:**

Upper extremity and tibiopedal arterial access are increasingly used during endovascular therapies. Balloon compression hemostasis devices in these anatomic locations have been described, but most utilize a compression surface extending well beyond the puncture site. We report single-center experience with an arterial puncture-focused compression device following upper extremity and tibiopedal access.

**Patients and Methods:**

A series of 249 focused compression hemostasis devices (VasoStat, Forge Medical, Bethlehem, Pennsylvania, USA) were used in 209 patients following lower extremity (*n* = 63) and upper extremity (*n* = 186; radial: 90%) arterial access procedures using 4–7 French sheaths. Demographic, operative, and follow-up data were collected. Logistic regression was used to evaluate potential association between patient/operative variables and time to hemostasis.

**Results:**

Primary hemostasis was achieved in 97.2% (242/249) following sheath removal; in 7 cases (2.8%) puncture site oozing occurred after initial device removal and required reapplication. Secondary hemostasis was 100% (249/249). Seven complications (2.8%) were recorded: 5 minor hematomas (2%) and 2 transient access artery occlusions (0.8%). Mean time to hemostasis enabling device removal was 55 ± 28 min. Elevated body mass index (BMI) was not associated with increased time to hemostasis (*p* = 0.31). Accessed artery, sheath size, and heparin dose were also not associated with time to hemostasis (*p* = 0.64; *p* = 0.74; *p* = 0.75, respectively).

**Conclusions:**

The focused compression hemostasis device enabled rapid hemostasis with a low complication rate. Time to hemostasis was independent of BMI, access site, sheath size, or heparin dose.

## Introduction

Upper extremity and tibiopedal arterial access for percutaneous angiography and intervention have become increasingly common. The benefits of radial artery access relative to common femoral artery access are well studied: decreased bleeding and vascular complications, increased patient comfort and satisfaction, decreased time to hemostasis, and in the setting of percutaneous coronary intervention for ST-elevation myocardial infarction, significantly lower mortality [1–4]. Radial access is also used during noncoronary interventions [5], also showing improved patient satisfaction [6–9]. Retrograde tibiopedal access offers several mechanical advantages in the setting of lower extremity revascularization and may allow for successful endovascular treatment of tibioperoneal and femoropopliteal disease when antegrade-only techniques have failed [10, 11].

Various hemostasis devices in clinical practice have been described following upper extremity and tibioperoneal arterial access, although published outcomes are generally limited to their indicated use for radial artery hemostasis. Band devices employing balloon compression or compressive plates such as the TR Band (Terumo, Somerset, NJ), SafeGuard Radial compression device (Merit Medical, South Jordan, UT), Zephyr Device (Advanced Vascular Devices, Milwaukie, OR), and the Radistop (Abbott Vascular, Santa Clara, CA) have been used to achieve hemostasis following radial artery access [12–15]. These devices apply broad (≥ 6 cm^2^ area of compression) force over the volar surface of the wrist and may inadvertently compress the ulnar artery or overly compress the radial artery, which are potential contributors to radial artery occlusion (RAO) and patient discomfort [16–18]. RAO has been reported in up to 8–31% of procedures using these devices for radial hemostasis when assessed by ultrasound [4, 19–23]. A randomized control trial of the TR Band and Radistop demonstrated early RAO rates of 8.9 and 9.6%, as well as long times to hemostasis of 5.3 and 4.8 h, respectively [13]. Moreover, achieving hemostasis after tibiopedal access, an approach useful in traversing infrainguinal stenoses in patients with critical limb ischemia, is not currently cleared by the Food and Drug Administration for most existing radial compression devices including the TR Band.

**Fig. 1:**
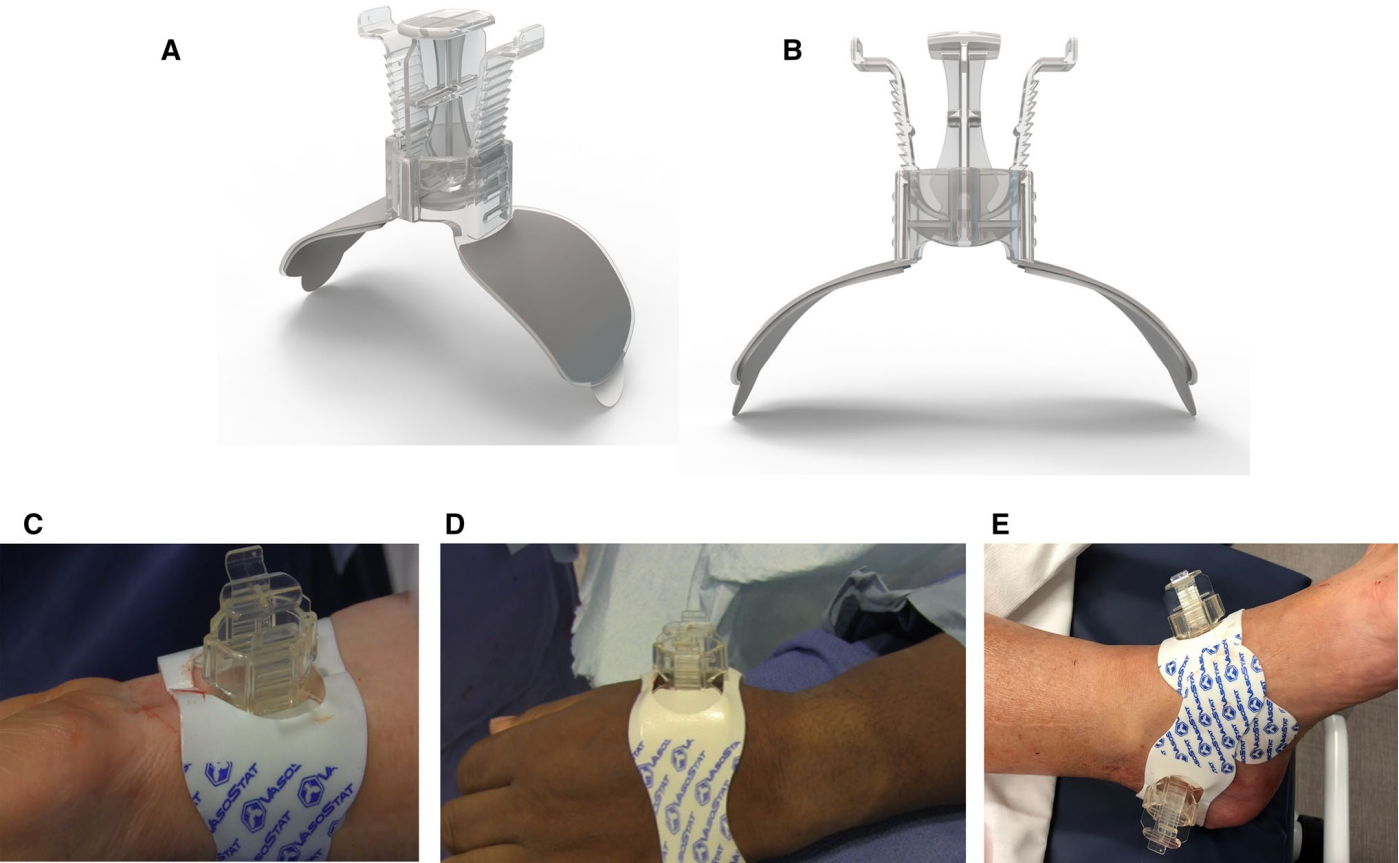
VasoStat Hemostasis Device. A central convex compression surface provides graded puncture site compression using a ratcheting mechanism incorporated within the base of the device. An overlying elastomeric adhesive pad further maintains alignment over the arterial access site. (**A**/**B** Isometric and front views of device, **C** radial, **D** distal radial, **E** combined posterior tibialis and dorsalis pedis)

The VasoStat hemostasis device (Forge Medical Inc., Bethlehem, PA), recently introduced in the United States and Japan, was developed to address several limitations of existing hemostasis devices by utilizing more focused (≤ 2 cm^2^ area of compression) and mechanically graded compression of the artery to achieve hemostasis (Fig. 1). The VasoStat device is FDA-cleared and CE-marked for hemostasis after both upper extremity and transpedal access, and its focused compression mechanism may lead to more rapid hemostasis compared to larger compression surfaces by band devices such as the TR Band [24–26]. This study investigated the use of the focused compression device following upper and lower extremity arterial access and assessed patient and operative variables influencing times to hemostasis.

## Patients and Methods

A prospectively maintained quality assurance database (Hi-IQ, Conexsys, Lincoln, RI) identified over a 36-month period 249 focused compression devices (VasoStat) used in 209 unique patients following upper and lower extremity arterial access performed during upper and lower extremity revascularization or visceral embolization procedures (Fig. 1). This study was retrospective and received institutional IRB exemption. Inclusion criteria were patients who underwent transpedal (anterior tibial, posterior tibial, or dorsalis pedis) or upper extremity (radial, ulnar, or brachial) arterial access followed by use of the VasoStat device to achieve hemostasis; the VasoStat is FDA-cleared and CE-marked for upper extremity arterial and tibioperoneal hemostasis. Patient demographic and operative data were collected; data were accessed and protected according to institutional protocols for retrospective clinical studies. Operators were attending interventional radiologists and interventional radiology fellows working at a single institution. In accordance with institutional practice, all accessed arteries were also evaluated prior to upper extremity and transpedal access using ultrasound to measure vessel diameter using electronic calipers. Ultrasound-guided micropuncture access was used in all patients. VasoStat use was according to the manufacturer’s instructions for use; patent hemostasis technique was used for all device applications. With the sheath still in place at the conclusion of the interventional procedure, the adhesive base of the device was aligned over clean and dry skin so that the aperture of the base was centered over the point of arterial entry. The base was then secured to the skin with gentle pressure applied over the adhesive wings. Next, the fenestrated elastomeric adhesive band was adhered over the aperture of the base and circumferentially applied around the upper or lower extremity. The compressive plunger of the device was inserted into the base of the device and progressive pressure applied through the integrated ratcheting mechanism to apply initial compression to the puncture site. Next, as the sheath was withdrawn additional 2–4 ratchet positions of compression (determined by patient anatomy) were applied with the device to achieve puncture site hemostasis. The pulse distal to the device was then verified with palpation and/or duplex ultrasound; if the pulse was weak or absent the compression was released by a single ratchet position to enable return of the pulse. This maneuver was repeated as needed in 1-ratchet increments to ensure patency of the accessed artery during device compression.

Age, sex, and BMI were recorded, as well as operative details including sheath size, heparin dose, fluoroscopy time (used as a proxy of procedural complexity), and time of hemostasis device placement. Time to hemostasis enabling device removal was documented in the electronic medical record (Epic, Verona, Wisconsin) by the interventional radiology nurses. Nurses assessed the puncture site for device removal beginning 45 min following VasoStat placement by uncoupling the central compressive plunger of the device by a single ratchet position. If no puncture site bleeding was observed, the plunger was uncoupled an additional ratchet position, the puncture site visually reassessed for hemostasis, and the device removed using gentle traction of the adhesive footplates. If puncture site oozing was observed, the compressive plunger was advanced a single ratchet position to stop bleeding, and the puncture site was reassessed for device removal following an additional 10–15 min.

Complications related to VasoStat application were documented using a prospectively maintained quality assurance database and confirmed by comparison with operative and IR clinic notes; patients were seen in the IR clinic within 30 days of each procedure during which the accessed artery was assessed with Doppler and/or duplex examination. Arterial diameter at each access site was measured using electronic calipers from ultrasound images in the picture archival and communication system (PACS) (Sectra AB, Linköping, Sweden).

### Statistical Analysis

Time to hemostasis and complications were compared between patient subsets based on BMI, heparin dose, sheath size, and arterial access site (upper extremity versus lower extremity). Patient subsets for BMI comparison were separated based by BMI < 30 kg/m^2^ or BMI ≥ 30 kg/m^2^. Subsets for heparin dose were heparin ≤ 3000 units (the median heparin dose) or heparin > 3000 units. Patient subsets for sheath size were ≤ 5 F and 6 F. Access site cohorts were divided based on transpedal versus upper extremity access (of which 90% were radial, and the remainder ulnar and brachial). Time to hemostasis was compared using Student’s *t* test and analysis of variance (ANOVA). Rates of complications were compared using Fisher’s exact test. Univariate and multivariate logistic regression was performed with Stata (Stata, College Station, TX) using the above cutoff values to explore association between these variables and time to hemostasis exceeding mean time for the entire patient cohort. A *p* value < 0.05 was considered the threshold of statistical significance.

## Results

In a 36-month period, a total of 186 upper extremity arterial access sites and 63 tibiopedal arterial access sites had hemostasis achieved with the VasoStat hemostasis device. Patient characteristics are listed in Table 1 and procedural details in Table 2. Mean access artery diameters ranged from 2.1 to 2.9 mm (excluding one peroneal artery measuring 3.9 mm) (Table 3). Six patients underwent radial artery access on the dorsum of the hand (in the anatomic snuffbox) with successful hemostasis achieved in all six cases. Mean time to hemostasis using the VasoStat device for the entire patient cohort was 55 min (S.D. ± 28 min). When stratified by sheath size, no significant differences in time to hemostasis were seen (Table 4). Five minor hematomas developed which did not require further intervention (2.0%), 7 instances of continued oozing occurred from the puncture site after premature device removal which were managed with VasoStat reapplication or supplemental manual compression until complete hemostasis was achieved (2.8%). In instances where continued puncture site oozing was noted, the cumulative time to hemostasis was utilized for analysis (after a another VasoStat was applied and subsequently removed).

**Table 1:** Patient characteristics

Patient characteristic	All patients (*n* = 249)
Age, mean (SD), year	60.0 (14.7)
BMI, mean (SD), kg/m^2^	30.6 (8.5)
Female sex, %	42.6

**Table 2:** Operative details

Operative details	(*n* = 249)
Heparin units, median, (25–75th IQR), range	3000, (3000–5000), (0–18,000)
Sheath size, F, (25–75th IQR), range	5, (5–5), (4–7)
Fluoroscopy time, min, mean (S.D.)	19.4 (15.9)
Time to hemostasis, min, mean (S.D.)	55 (28)

*S.D.* standard deviation

**Table 3:** Artery dimensions, mean (SD)

Artery	Number accessed	Diameter (mm)	Time to hemostasis (min) ± S.D.	*p* value
Radial (volar wrist)	162	2.5 (0.5)	54 ± 21	0.37^a^
Distal radial (snuffbox)	6	2.5 (0.2)	47 ± 8	
Ulnar	4	2.1 (0.9)	47 ± 18	
Brachial	14	2.9 (0.9)	68 ± 44	
Anterior tibialis	8	2.4 (0.6)	41 ± 9	
Dorsalis pedis	31	2.3 (0.5)	56 ± 26	
Posterior tibialis	23	2.6 (0.6)	46 ± 20	
Peroneal	1	3.9	47	^b^

^a^Analysis of Variance (ANOVA)

^b^Requires more than two observations for analysis

**Table 4:** Time to hemostasis by sheath size

Sheath size (French)	Number	Time to hemostasis (min) ± S.D.	*p* value
4	52	63 ± 26	0.39^a^
5	152	51 ± 22	
6	44	49 ± 19	
7	1	92	

^a^*t* test combining 4/5 French versus 6/7 French to account for group size heterogeneity

Two cases of access artery occlusion developed within 30 days (0.8%). Both cases of occlusion involved the radial artery and presented without symptoms at routine 2 week IR clinic visits; radial artery occlusion was diagnosed with duplex ultrasound. In both patients, the radial artery recanalized (with duplex ultrasound confirmation) within an additional 30 days of puncture site follow-up after one patient received a 2-week course of rivaroxaban (Xarelto, Johnson & Johnson, New Brunswick, NJ) and the other patient received three weeks of aspirin and clopidogrel; no further intervention was required.

Patient cohorts were divided by BMI, access site, sheath size, and heparin dose; no significant differences were found between time to hemostasis in the subsets analyzed (Table 5). Univariate logistic regression was performed to identify interactions between patient and operative characteristics and longer time to hemostasis (time to hemostasis greater than the cohort mean of 55 min) (Table 6). Cutoff points for logistic regression were determined based on mean and median values for age, BMI, heparin dose, and fluoroscopy time. To explore whether interaction existed between covariates to predict longer times to hemostasis, multivariate logistic regression was performed using mean time to hemostasis threshold of 55 min. No variable was associated with longer times to hemostasis (*p* = 0.64, results not shown).

**Table 5:** Patient subset times to hemostasis

Patient characteristic	Mean time to hemostasis (min)	*p* value
BMI (< 30/≥ 30)	57/53	0.31
Access site (Lower extremity/upper extremity)	53/56	0.62
Sheath size (≤ 5/6)	56/53	0.74
Heparin (≤ 3000/> 3000 units)	56/54	0.75

**Table 6:** Univariate logistic regression analysis

Variable	Odds ratio	Standard error	*p* value	Confidence interval (95%)
Age (> 60 years)	1.5	0.48	0.25	0.77–2.8
BMI (> 30 kg/m^2^)	0.66	0.22	0.21	0.34–1.3
Male/female (F)	0.95	0.32	0.89	0.50–1.8
Access site (upper versus lower)	1.4	0.57	0.39	0.64–3.1
Sheath size (> 5 F)	1.8	0.27	0.22	0.71–4.4
Heparin (> 3000 units)	0.77	0.28	0.48	0.38–1.6
Fluoroscopy time (> 20 min)	0.82	0.28	0.56	0.42–1.6

## Discussion

Upper extremity and tibiopedal arterial access provide significant patient and operator benefits during percutaneous angiography and intervention. Transpedal access has been increasingly utilized in lower extremity revascularization during treatment of femoral–popliteal and tibioperoneal disease [10, 11], and advantages of radial artery access for percutaneous coronary and peripheral intervention are well described [1–9]. Both access methods facilitate reduction in access site complications and time to patient ambulation; refined protocols for achieving hemostasis at these access sites may enable further improvement in patient safety and comfort to meet increasing demand for arterial access through these approaches. The hypothesis of the present study was that the focused, graded compression mechanism employed by the VasoStat is differentiated from existing devices and may thereby decrease time to hemostasis relative to prior devices used for upper extremity and transpedal hemostasis, and enable hemostasis spanning a spectrum of body habitus, access location, arteriotomy size, and heparin doses.

Mean times to hemostasis in a randomized trial by Rathore et al. comparing the TR Band (*n* = 395) and Radistop (*n* = 395) were 5.3 and 4.8 h, respectively. The authors reported early RAO rates at discharge of 8.9 and 9.6% for the TR Band and Radistop, decreasing to 5.6 and 8.0% chronic RAO at follow-up (ranging 4–6 months after intervention). Hematoma rates for the TR Band and Radistop were 5.4 and 2.2%, while oozing at the arteriotomy site occurred in 6.1 and 7.1%, respectively [13]. Another prospective study comparing the TR Band and HemoBand (HemoBand, Portland, OR) found the TR Band led to 4.4% early RAO (*n* = 250), compared to 11.2% (*n *= 250) of HemoBand subjects at 24 h. Late RAO documented at 30 days was 3.2 and 7.2% of TR Band and HemoBand patients, respectively [12]. More recent studies have reported shorter times to hemostasis as well as lower rates of RAO. In a randomized trial comparing the TR Band and SafeGuard Radial, Sanghvi et al. observed hemostasis times of 132 min (*n* = 155) and 141 min (*n* = 159) with rates of acute RAO of 3.8% and 6.3%, respectively [15].

Retrospective analysis of TR Band placement following transpedal access has indicated this application appears safe and effective, but times to hemostasis were not documented; additionally, tibioperoneal utilization of the TR Band is not cleared by the Food and Drug Administration [25]. The current series observed a mean time to hemostasis of 55 min with the VasoStat, with low rates of hematoma (2%), oozing after removal (2.8%), early access artery occlusion (0.8%), and late access artery occlusion (0%) following both radial, distal radial (anatomic snuffbox), brachial, ulnar, and tibioperoneal access. The short hemostasis times of the VasoStat may be attributed to the convex shape of the compression surface and the focused pressure over the entry point of the artery to enable efficient platelet plug formation. The mechanism of the device is designed to emulate that of manual compression, which has been shown to produce hemostasis faster than balloon compression devices [27].

Increased duration of radial artery compression following intervention has also been shown to increase rates of early and chronic radial artery occlusions, suggesting devices that achieve shorter times to hemostasis could potentially reduce instances of RAO [28]. Prospective trials including the PROPHET study documented increased rates of RAO following occlusive radial artery compression for hemostasis versus non-occlusive compression, identifying occlusive compression as an independent predictor of RAO [17, 18]. Larger sheath size has also been identified as increasing local complication rate and RAO [21]. Reduction in time to hemostasis using the VasoStat, resulting in decreased compression time, may account for the low rates of transient radial artery occlusion (0.8%) and absence of long-term radial artery occlusion (0%) observed in this study. Graded compression employed by the VasoStat enables maintained patency of the radial artery during utilization, which may also contribute to a reduction of RAO. No association between larger sheath size and local complications/increased RAO was seen with use of the VasoStat.

The current study found that body habitus, arterial access size (using the surrogate variable of sheath size), and heparin dose were not associated with time to hemostasis or complication rates in patients undergoing arterial access compression by the VasoStat device. Biederman et al. reported their experience with radial access among patients with morbid obesity (BMI ≥ 40 kg/m^2^) and achieved hemostasis in all 22 procedures performed in 17 patients using the TR Band. They did not report times to hemostasis but noted that balloon deflation began 60–90 min following band application [29]. In the present study, 20 patients had BMI exceeding 40 kg/m^2^ (range 41–73 kg/m^2^); mean time to hemostasis enabling VasoStat removal in this subset was 51 min, similar to the mean of 55 min for the entire cohort.

Transpedal access through the tibial, dorsalis pedis, and peroneal arteries was not associated with increased time to hemostasis or complication rate relative to radial or ulnar access. While rare events of access site pseudoaneurysm have been previously documented in the use of the TR Band and VasoStat devices for transpedal access [25], no pseudoaneurysms were observed in this series.

Limitations of this study include its single-center design, limited sample size, and comparison to historical controls as opposed to contemporaneous controls from data collected at the same institution. Accessed arteries were not evenly distributed among anatomic areas, reflective of a retrospective clinical cohort. Patient follow-up was variable, and it is possible that late occlusions or pseudoaneurysms could have occurred in some patients without detection. Activated clotting time (ACT) was not utilized as a threshold to determine time for sheath removal and VasoStat placement, as all sheaths were removed at the time of procedure completion. Times to hemostasis used in this study were based on clinical staff documentation in the medical record; delays between achieving hemostasis and documentation of hemostasis in the medical record may have occurred and thereby overestimated some actual times to hemostasis.

## Conclusions

The focused compression hemostasis device used in upper extremity and tibioperoneal access was associated with rapid hemostasis and low complication rates; shorter time to hemostasis may contribute to low observed rates of early and late access artery occlusion. Time to hemostasis was independent of patient age, sex, BMI, access site, sheath size, or heparin dose.
